# Integrated safety analysis of phase 3 studies for investigational microbiome therapeutic, SER-109, in recurrent CDI

**DOI:** 10.1017/ash.2023.281

**Published:** 2023-09-29

**Authors:** Matthew Sims, Charles Berenson, Stuart Cohen, Elaine Wang, Elizabeth Hohmann, Richard Nathan, Alberto Odio, Paul Cook, Kelly Brady, David Lombardi, Asli Memisoglu, Ananya De, Brooke Hasson, Bret Lashner, Louis Korman, Doria Grimard, Juan Carlos Moises Gutierrez, Barbara McGovern, Lisa Von Moltke

## Abstract

**Background:** *Clostridioides difficile* infection (CDI) often recurs in patients aged ≥65 years and those with comorbidities. Clinical trials often exclude patients with history of immunosuppression, malignancy, renal insufficiency, or other comorbidities. In a phase 3 trial (ECOSPOR III), SER-109 was superior to placebo in reducing recurrent CDI (rCDI) risk at week 8 and was well tolerated. We report integrated safety data for SER-109 in a broad patient population through week 24 from phase 3 studies: ECOSPOR III and ECOSPOR IV. **Methods:** ECOSPOR III was a double-blind, placebo-controlled trial conducted in participants with ≥2 CDI recurrences randomized 1:1 to placebo or SER-109. ECOSPOR IV was an open-label, single-arm study conducted in 263 patients with rCDI enrolled in 2 cohorts: (1) rollover participants from ECOSPOR III with on-study recurrence and (2) participants with ≥1 CDI recurrence, inclusive of the current episode. In both studies, the investigational product was administered as 4 oral capsules over 3 days. Treatment-emergent adverse events (TEAEs) were collected through week 8; serious TEAEs and TEAEs of special interest (ie, bacteremia, abscess, meningitis) were collected through week 24. **Results:** In total, 349 participants received SER-109 in ECOSPOR III and/or ECOSPOR IV (mean age 64.2; 68.8% female). Chronic diseases included cardiac disease (31.2%), immunocompromised or immunosuppressed (21.2%), diabetes (18.9% ), and renal impairment or failure (13.2%). Overall, 221 (63.3%) of 349 participants who received SER-109 experienced TEAEs through week 24. Most were mild to moderate and gastrointestinal. The most common (>5% of participants) treatment related TEAEs were flatulence, abdominal pain and distension, decreased appetite, constipation, nausea, fatigue, and diarrhea. No participants experienced a treatment-related TEAE leading to study withdrawal. Invasive infections were observed in 28 participants (8%); those with identified pathogens were unrelated to SER-109 species, and all were deemed unrelated to treatment by the investigators. There were 11 deaths (3.2%) and 48 participants (13.8%) with serious TEAEs, none of which were deemed treatment related. There were no clinically important differences in the safety profile across subgroups of sex, race, prior antibiotic regimen, or number of CDI recurrences. No safety signals were observed in participants with renal impairment or failure, diabetes, cardiac disease, or immunocompromised or immunosuppressed individuals. **Conclusions:** In this integrated analysis of phase 3 trials, SER-109, an investigational microbiome therapeutic, was well tolerated in this vulnerable patient population with prevalent comorbidities. No infections, nor those with identified pathogens, were attributed to SER-109 or product species. This safety profile might be expected because this purified product is composed of spore-forming Firmicutes normally abundant in the healthy microbiome.

**Financial support:** This study was funded by Seres Therapeutics.

**Disclosures:** None

